# A Nested PCR-Based Point of Care Testing of Multiplex Pathogens Associated with Bloodstream Infection

**DOI:** 10.3390/pathogens15020211

**Published:** 2026-02-13

**Authors:** Shihao Jiao, Juntao Meng, Jianing Wu, Duoxiao Zhang, Xingyu Liu, Zhiqiang Han, Yuxin Wang, Shijue Gao, Zijin Zhao, Yujie Xiang, Junkai Ren, Qian Ma, Xinxin Li, Xinxin Shen, Xuejun Ma, Yanqing Tie

**Affiliations:** 1 Graduate School, North China University of Science and Technology, Tangshan 063210, China; 2 Department of Clinical Laboratory, Hebei General Hospital, Shijiazhuang 050051, China; 3 Hebei Key Laboratory of Molecular Medicine, Shijiazhuang 050051, China; 4 Hebei Clinical Research Center for Laboratory Medicine, Shijiazhuang 050051, China; 5 National Key Laboratory of Intelligent Tracking and Forecasting for Infectious Diseases, NHC Key Laboratory of Medical Virology and Viral Diseases, National Institute for Viral Disease Control and Prevention, Chinese Center for Disease Control and Prevention, Beijing 102206, China; 6 Graduate School, Hebei North University, Zhangjiakou 075000, China; 7 Graduate School, Hebei Medical University, Shijiazhuang 050031, China

**Keywords:** *Escherichia coli*, *Klebsiella pneumoniae*, *Streptococcus pneumoniae*, bloodstream infection, point-of-care testing, multiple detection, nested PCR

## Abstract

Bloodstream infections (BSI) carry high mortality, but traditional blood culture is too slow for urgent clinical needs. This study aims to develop a rapid point-of-care testing assay based on one-tube multiplex nested polymerase chain reaction (PCR) (OM-NPCR-POCT) for early diagnosis of three pathogens in bloodstream infection patients: *Escherichia coli* (ECO), *Klebsiella pneumoniae* (KPN), and *Streptococcus pneumoniae* (SPN). The analytical sensitivity of the one-tube multiplex nested PCR (OM-NPCR) was evaluated using recombinant bacterial plasmids. The analytical sensitivity of the OM-NPCR-POCT assay was assessed using simulated samples. The clinical performance was evaluated in 90 clinical blood samples, with results compared to conventional quantitative PCR (qPCR). Finally, the agreement between the two detection methods was assessed via Kappa analysis. The limits of detection (LODs), calculated from serial dilution experiments, were 4, 2, and 1 copies/μL for plasmids ECO, KPN, and SPN, respectively. The OM-NPCR-POCT assay achieved LODs of 20, 10, and 10 CFU/mL for ECO, KPN, and SPN in simulated samples, with a total testing time of approximately 2 h. The clinical evaluation of OM-NPCR-POCT demonstrates consistency with conventional qPCR while exhibiting higher sensitivity. This method has potential as a rapid diagnostic tool for early bloodstream infection detection.

## 1. Introduction

Bloodstream infections (BSI) refer to a systemic, disseminated infection caused by pathogenic microorganisms (including bacteria, fungi, viruses, and protozoa) or their toxic products that invade the bloodstream. If not promptly and effectively controlled, BSI can progress to sepsis, septic shock, disseminated intravascular coagulation (DIC), and multiple organ failure, resulting in extremely high mortality rates and constituting a significant global public health burden [1]. Recent epidemiological studies estimate that global deaths attributable to sepsis reached 21.4 million in 2021, with approximately 2.9 million occurring in children under five years of age. Both the incidence rate and associated healthcare costs continue to rise [2]. Studies indicate that for every hour of delayed treatment for bloodstream infection, patient mortality increases by approximately 7.8% [3]; a 6 h delay results in a mortality rate of 58% [4]. Epidemiological data indicate that *Escherichia coli* (ECO) is the leading cause of bloodstream infections, accounting for approximately 20–27% of cases. *Klebsiella pneumoniae* (KPN) is another major pathogen, particularly associated with healthcare-associated infections, representing approximately 4.8–13.2% of bloodstream isolates and drawing significant public health attention due to its propensity for multidrug resistance [5]. *Streptococcus pneumoniae* (SPN) remains a key Gram-positive pathogen in community-acquired bloodstream infections, especially in pneumonia-associated sepsis, contributing to approximately 4% of cases [6]. Together, these pathogens represent a considerable share of the clinical burden of bloodstream infections across both community and healthcare settings.

Blood culture remains the gold standard for diagnosing bloodstream infections. The standard procedure is time-consuming, with the entire cycle taking up to 72 h. Concurrently, blood culture positivity rates are influenced by multiple factors, including blood volume, collection timing, sampling techniques, and operator proficiency, posing risks of false negatives. Sensitivity remains as low as 67.7% [7]. Thus, current blood culture methods fail to meet clinical demands. Early and accurate diagnosis, coupled with appropriate antimicrobial therapy, is critical for improving BSI patient outcomes and reducing mortality [8].

Emerging nucleic acid point-of-care testing (POCT) technologies integrate molecular biology detection with point-of-care diagnostic platforms, consolidating nucleic acid extraction, amplification, and fluorescence detection within a single system. Characterized by portability, ease of operation, rapid reporting, and relatively lower costs [9,10], these technologies are well-suited for resource-constrained field or primary care settings. However, most current nucleic acid POCT methods still lag behind traditional laboratory methods in sensitivity, failing to fully meet the demands for precise diagnosis of severe infections. Consequently, there is an urgent need for a POCT detection technology that combines rapid response, high sensitivity, and high specificity [11].

In our previous research, comparable sensitivity of specificity was observed between the one-tube nested quantitative real-time polymerase chain reaction (PCR) assay and the traditional two-step nested method. For bacterial detection, this method achieved a sensitivity of a single copy per reaction for *Bordetella pertussis* infection, which is 100 times the sensitivity of the conventional PCR detection (100 copies per reaction) [12]. In viral detection, its limit of detection for respiratory syncytial virus is 1.02 × 10^−1^ TCID50/mL, representing 25 times the sensitivity of real-time PCR [13]. Additional studies have detected *Brucella* DNA in human blood using a one-tube nested quantitative PCR (qPCR) approach, achieving an analytical sensitivity of 100 fg/μL—100 times higher than conventional qPCR [14]. These prior investigations demonstrate nested PCR’s feasibility for clinical samples, offering superior sensitivity compared to traditional qPCR. However, all these reports remain constrained by manual operational steps.

In this study, we developed a point-of-care testing assay based on one-tube multiplex nested polymerase chain reaction (OM-NPCR-POCT) detection method targeting common bloodstream infection pathogens ECO, KPN, and SPN. This approach leverages the high sensitivity of nested PCR and the convenience of rapid, fully automated nucleic acid extraction and fluorescence detection via POCT instruments. The method encompasses both one-tube multiplex nested PCR (OM-NPCR) protocol establishment and POCT system integration debugging. The total turnaround time is 2 h, enabling direct detection of three pathogens—ECO, KPN, and SPN—from blood without culture. Performance was further validated using simulated quantitative blood samples and clinical samples and compared with qPCR methods, demonstrating the OM-NPCR-POCT’s efficacy for rapid and accurate detection of these three common pathogens associated with bloodstream infection.

## 2. Materials and Methods

### 2.1. Sample Collection and Nucleic Acid Extraction

Reference strains ECO (ATCC 25922), SPN (ATCC 49619), and KPN (ATCC 11296) were provided by the National Institute for Communicable Disease Control and Prevention, Chinese Center for Disease Control and Prevention. A total of 90 clinical blood samples were collected by the Microbiology Laboratory of the Clinical Laboratory at Hebei Provincial People’s Hospital, from 26 April to 24 October 2025, including 20 KPN-positive blood cultures, 20 ECO-positive blood cultures, 5 SPN-positive blood cultures, and 45 blood samples with negative blood cultures. Blood culture-positive bacteria underwent matrix-assisted laser-desorption/ionization time of flight mass spectrometry (MALDI-TOF MS) species identification, with EDTA-anticoagulated whole blood collected concurrently. Clinical samples were transported on ice to the Central Laboratory of the Chinese Center for Disease Control and Prevention (China CDC) Institute for Viral Disease Control and Prevention and stored at −20 °C. Nucleic acids from bacterial strains were extracted using the FastPure^®^ Microbial DNA Extraction Kit (Vazyme, Nanjing, China) according to the manufacturer’s protocol. Non-POCT fluorescence detection was performed using the Archimedes real-time fluorescent quantitative PCR system (Rocgene, Beijing, China).

### 2.2. Design of Nested Primers and Probes and Plasmid Construction

Complete pathogen gene sequences for ECO (ybbw), SPN (lytA), and KPN (khe) were downloaded from the National Center for Biotechnology Information (NCBI). BioEdit7.0.9 software was used for sequence alignment to identify highly conserved regions suitable for primer design, meeting species-level identification requirements. Each target required one pair of outer primers, one pair of inner primers, and one TaqMan probe for nested PCRs. Primers for ECO, KPN, and SPN were designed using Primer6.0 and Oligo7.60 software with efficacy further validated via Primer-BLAST on the NCBI website (http://www.ncbi.nlm.nih.gov/BLAST/, accessed on 8 January 2026). Outer primers were designed with a Tm value approximately 10 °C higher than inner primers. qPCR primer-probe combinations were selected based on published literature [15,16,17]. All primers and probes were synthesized by BiOligo Biotechnology (Shanghai, China) Co., Ltd. ybbw, khe, and lytA gene fragments (308 bp, 310 bp, and 306 bp, respectively) from ECO, KPN, and SPN were cloned into the pUC57 vector provided by TsingKe Biotech (Beijing, China). Recombinant plasmid DNA was quantified using the Qubit^®^ dsDNA HS Assay Kit (Thermo Fisher Scientific, Waltham, MA, USA) and the Qubit 2.0 Fluorometer (Life Technologies, Carlsbad, CA, USA). The plasmid copy number was calculated using the following formula: Plasmid copy number (copies/μL) = [6.02 × 10^23^ × plasmid concentration (ng/μL) × 10^−9^]/[plasmid length × 660]. Finally, standard plasmids were prepared in 1 × TE buffer with a 10-fold concentration gradient, covering a range from 100 to 10^5^ copies/μL. All plasmids were stored at −20 °C until use.

### 2.3. Establishment and Optimization of the OM-NPCR Reaction System

Based on our laboratory’s prior research, nested PCR was performed in one tube through two stages: the first stage involves 10 cycles with the outer primers, followed by 40 cycles with the inner primers in the second stage [12]. To determine optimal reaction conditions and identify suitable working temperatures for both inner and outer primers, gradient PCR was conducted at temperatures ranging from 52 °C to 74 °C. Following the determination of temperature conditions, primer concentrations were repeatedly adjusted to optimize the final reaction parameters. All primers and probes are listed in Table 1. Probes for ECO, KPN, and SPN were labeled with CY5, FAM, and VIC fluorophores, respectively, enabling simultaneous detection of bloodstream infection bacteria within the OM-NPCR reaction system.

The total reaction volume for OM-NPCR was 20 μL, comprising 10 μL 2× Taq Pro U+ Multiple Probe qPCR Mix (Vazyme, Nanjing, China), 0.2 μL of each outer primer (5 μM), 0.4 μL of each inner primer (10 μM), 0.2 μL each of the ECO and KPN TaqMan probes (10 μM), 0.12 μL of the SPN TaqMan probe, 4.88 μL of DEPC-treated water, and 1 μL of DNA template. The OM-NPCR reaction program was as follows: 95 °C for 3 min (1 cycle); 95 °C for 15 s, 70 °C for 45 s (10 cycles); 95 °C for 15 s, 60 °C for 45 s with fluorescence acquisition (40 cycles).

### 2.4. Sensitivity, Specificity, and Repeatability Analysis of the OM-NPCR Detection Method

The sensitivity of OM-NPCR was evaluated using recombinant plasmids at concentrations ranging from 10^0 to^ 10^5^ copies/μL. DEPC-treated water served as the negative control for each test. The method’s sensitivity and reproducibility were evaluated by performing eight tests at each concentration over different time points and the results were compared with qPCR. Additionally, 15 other BSI-associated pathogens were used to evaluate the specificity, including: *Streptococcus agalactiae*, *Streptococcus pyogenes*, *Listeria monocytogenes*, *Staphylococcus aureus*, *Pseudomonas aeruginosa*, *Neisseria meningitidis*, *Enterococcus faecalis*, *Enterococcus faecium*, *Enterobacter cloacae*, *Proteus mirabilis*, *Pseudomonas maltophilia*, *Candida tropicalis*, *Candida kruesi*, *Candida glabrata*, and *Candida albicans*. These pathogens were passaged in culture, mixed with blood from healthy individuals, and nucleic acids were manually extracted using the FastPure^®^ Microbiome DNA Isolation Kit (Vazyme, Nanjing, China). Finally, the extracted nucleic acids were detected using OM-NPCR assay.

### 2.5. Detection Sensitivity of Conventional qPCR

To compare the detection sensitivity between OM-NPCR and conventional qPCR [18], recombinant plasmids and simulated clinical samples for ECO, KPN, and SPN were used for qPCR detection. The total qPCR reaction volume was 20 μL, comprising 10 μL 2× Taq Pro U+ Multiple Probe qPCR Mix, 0.4 μL internal primer (10 μM), 0.2 μL TaqMan probe (10 μM), 8 μL DEPC-treated water, and 1 μL DNA template. The amplification process was as follows: 95 °C for 3 min (1 cycle); 95 °C for 15 s, 60 °C for 45 s with fluorescence acquisition (40 cycles).

### 2.6. Preparation of Quantitative Simulated Blood Samples

Strains of ECO, KPN, and SPN that had been stored at −80 °C were thawed and revived at room temperature. Each strain was inoculated onto Columbia blood agar plates, which were incubated in a 5% CO_2_ incubator at 37 °C for 24 h. After two passages, the third-generation reference strains were used for testing. First, we picked a single colony with an inoculation loop and inoculated it into Brain Heart Infusion (BHI) medium. This was incubated overnight at 37 °C on a shaking incubator at 220 rpm. The next day, we took 1 mL of the bacterial suspension, centrifuged at 5000 *g* for 3 min, discarded the supernatant, and resuspended in 1 mL of PBS. We repeated this process twice to achieve a seven-fold dilution series (labeled 10^−1^ to 10^−6^). We divided a blood agar plate into three columns, each with three rows (nine zones total). We assigned one column per bacterial concentration, added 10 μL to each zone and repeated three times. We recorded colony counts and calculated the original concentration from the average. This yielded a relatively accurate quantification. Subsequently, bacterial suspensions at concentrations of 5, 10, 20, 50, 100, 200, 500, and 1000 CFU were added to 1 mL of sterile healthy human whole blood samples to prepare simulated samples (final concentrations: 5, 10, 20, 50, 100, 200, 500, and 1000 CFU/mL).

### 2.7. Evaluation of Manual Detection and POCT Instrument Detection for Simulated Samples

Manual nucleic acid extraction was performed using a nucleic acid extraction kit on three bacterial blood simulant samples, followed by detection via OM-NPCR and qPCR techniques. Each experiment was repeated three times.

**Figure 1:**
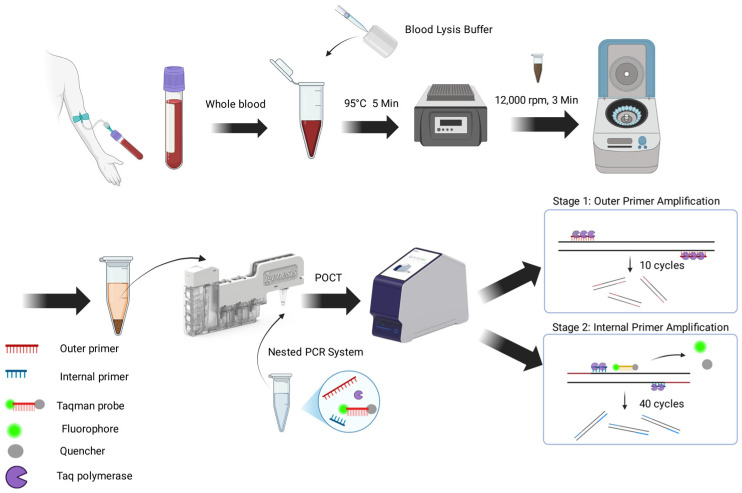
Schematic Diagram of OM-NPCR-POCT Assay.

For OM-NPCR-POCT detection, simulated blood samples containing varying concentrations of ECO, KPN, and SPN were mixed with Blood lysis buffer (Vazyme, Nanjing, China) at a 1:1 ratio. They were vortexed and heated at 95 °C for 5 min to ensure complete cell lysis and promote hemoglobin precipitation with components in the lysis buffer. Subsequently, samples were centrifuged at 12,000 rpm for 3 min, and the supernatant was collected for subsequent use. Using the molecular POCT instrument (ENucFlow-PS2, fully automated closed nucleic acid extraction and detection analysis system) from Auron Biotechnology Co., Ltd. (Shanghai, China), we added 400 μL of supernatant as the reaction template to the sample well of the kit. The OM-NPCR and qPCR systems were respectively added to the PCR reaction tubes of the cartridge for separate detection. The detection principle of OM-NPCR-POCT is illustrated in Figure 1. Each experiment was repeated three times. The results were compared to evaluate the minimum detection limits of both methods.

### 2.8. Evaluation of Clinical Blood Sample Detection Performance

EDTA-anticoagulated whole blood samples from patients with positive blood culture results and negative blood culture samples were tested using both OM-NPCR-POCT and manual qPCR. The consistency between the two detection methods was compared to assess their clinical performance. Results were analyzed using IBM SPSS Statistics version 25 software. Kappa analysis was employed to compare the consistency between the OM-NPCR-POCT and manual qPCR. A Kappa coefficient closer to 1 indicates better agreement. *p* < 0.05 was considered statistically significant.

## 3. Results

### 3.1. Establishment and Optimization of the OM-NPCR Reaction System

The ratios of different primers and probes in OM-NPCR were adjusted to ensure efficient amplification of each target. The optimal primer concentrations for ECO, KPN, and SPN targets were 0.2 μM, while probe concentrations were set at a 5:5:3 ratio. Temperature gradient testing and PCR products electrophoresis were conducted using the plasmid as the reaction template, 70 °C, was identified as an optimal temperature under which the inner primers did not amplify during the outer primer amplification phase for all three bacteria.

### 3.2. Sensitivity, Specificity, and Repeatability Analysis of the OM-NPCR Detection Method

**Figure 2:**
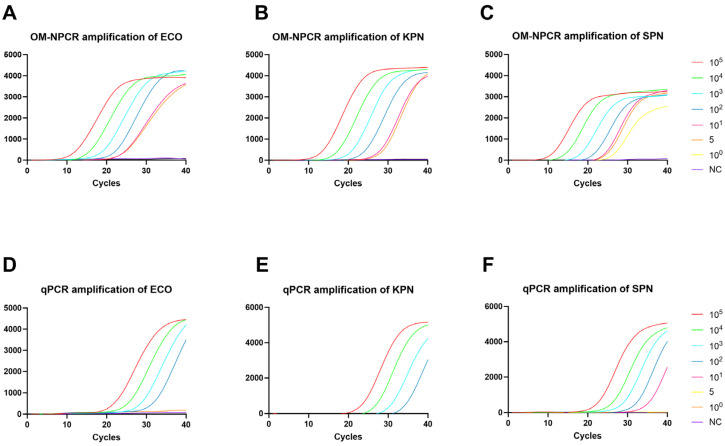
Sensitivity analysis for OM-NPCR, and qPCR was performed using recombinant plasmids at 10^0^–10^5^ copies/μL. ECO (**A**), KPN (**B**), and ECO (**C**) recombinant plasmids were amplified by OM-NPCR; ECO (**D**), KPN (**E**), and SPN (**F**) recombinant plasmids were amplified using qPCR. NC, negative control; ECO, *Escherichia coli*; KPN, *Klebsiella pneumoniae*; SPN, *Streptococcus pneumoniae*.

The sensitivity of OM-NPCR for detecting ECO, KPN, and SPN was evaluated using recombinant plasmids. Probability analysis of the detection results indicated 95% detection limits of 4 copies/μL, 2 copies/μL, and 1 copies/μL for the ECO, KPN, and SPN plasmids, respectively (Figure 2A–C). Results from eight replicate tests for each recombinant plasmid concentration are presented in Table S1. No amplification curves were observed in negative controls, and replicate test results were consistent.

Further detection in the simulated blood samples confirmed the excellent specificity of OM-NPCR, yielding positive results only for ECO, KPN, and SPN, with no cross-reactivity observed against 15 other common bloodstream infection pathogens. Furthermore, no fluorescent amplification signals were detected in any of the negative controls, further validating the specificity and reliability of OM-NPCR. Results are shown in Table S2.

### 3.3. Detection Sensitivity of Conventional qPCR

The sensitivity of qPCR for plasmid ECO, KPN, and SPN 10^2^, 10^2^ and 10 copies/μL (Figure 2D–F). Sensitivity comparison results demonstrate that the OM-NPCR assay exhibits significantly superior detection capabilities compared to conventional qPCR. In plasmid detection, the sensitivity enhancement multiples of OM-NPCR for different pathogens are as follows: ECO 25-fold, KPN 25-fold, SPN 10-fold. When detecting low-concentration plasmids, OM-NPCR amplification curves outperformed qPCR (Figure 2A–C).

### 3.4. Evaluation of Manual Detection and POCT Instrument Detection for Simulated Samples

**Figure 3:**
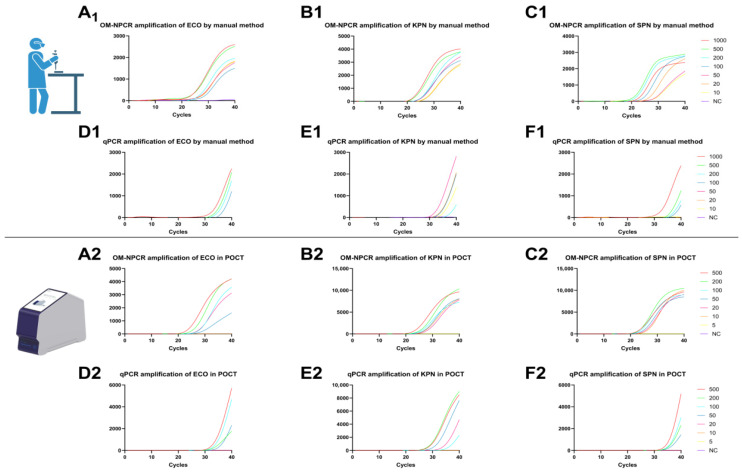
Manual extraction of nucleic acids from simulated blood samples (10–1000 CFU/mL) for LOD detection by OM-NPCR and qPCR. OM-NPCR detection of ECO, KPN, and SPN (**A1**–**C1**). qPCR detection of ECO, KPN, and SPN (**D1**–**F1**). Simulated blood samples (5–500 CFU/mL) were tested using the POCT platform by performing OM-NPCR and qPCR assays for the detection of ECO, KPN, and SPN. OM-NPCR was used to detect ECO, KPN, and SPN (**A2**–**C2**), while qPCR detected ECO, KPN, and SPN (**D2**–**F2**). CFU, colony-forming units; NC, negative control; ECO, *Escherichia coli*; KPN, *Klebsiella pneumoniae*; SPN, *Streptococcus pneumoniae*.

For manual qPCR, we employed the FastPure^®^ Microbial DNA Extraction Kit to extract nucleic acids from simulated blood samples at varying concentrations. The limits of detection (LODs) for OM-NPCR detection of ECO, KPN, and SPN blood simulants were 10, 10, and 10 CFU/mL, respectively (Figure 3(A1–C1)). In contrast, qPCR detection yielded LODs of 100, 50, and 100 CFU/mL, respectively. (Figure 3(D1–F1)).

The OM-NPCR-POCT assay demonstrated detection limits of 20, 10, and 10 CFU/mL for ECO, KPN, and SPN blood simulant samples, respectively (Figure 3(A2–C2)). In contrast, qPCR yielded detection limits of 50, 20, and 50 CFU/mL for the same samples (Figure 3(D2–F2)).

### 3.5. Evaluation of Clinical Blood Sample Detection Performance

A retrospective analysis was conducted on 90 blood samples (45 culture-positive and 45 culture-negative) using OM-NPCR-POCT and qPCR. Clinical sample results are presented in Table 2, showing discrepancies in 6 samples. In cases where OM-NPCR-POCT is positive, qPCR is negative. Kappa values for ECO, KPN, and SPN were 0.910, 0.877, and 0.852, respectively, all with *p* values < 0.05 indicating statistical significance. The two assays showed consistency, but the OM-NPCR assay was more sensitive than qPCR. All negative controls in the OM-NPCR-POCT run yielded negative results.

## 4. Discussion

Among existing technologies for the direct detection of blood samples, quantitative polymerase chain reaction (qPCR) is regarded as a highly promising technical approach due to its high sensitivity and relatively short detection cycle. Building upon this foundation, multiple detection protocols have been developed, including multiplex qPCR, PCR-NMR hybridization, and droplet digital PCR [19,20,21]. When applied in clinical and experimental settings, these methods have been reported to achieve high sensitivity and specificity while significantly reducing reporting time. However, they still face notable limitations: their detectable pathogen spectrum remains relatively limited, they demand stringent laboratory environments and operational conditions, and their overall costs remain high, imposing significant burdens on healthcare institutions and patients [22].

POCT has experienced rapid development in the medical field. Over the past five years, nucleic acid testing systems, as disruptive innovations, have propelled POCT to capture over 30% of the in vitro diagnostics market share [23]. Molecular biology POCT devices (such as the GeneXpert system) utilize automated real-time PCR to directly detect pathogen nucleic acids—including drug resistance genes like MRSA—from clinical samples within approximately one hour. Primarily employed for rapid identification of specific pathogens in respiratory specimens and wound swabs, these technologies demonstrate the technical feasibility of direct, rapid pathogen detection. However, their application remains relatively limited in the critical field of bloodstream infection diagnosis. Current POCT methods for managing sepsis in intensive care units (ICUs) primarily detect host response biomarkers to infection—IL-6, IL-10, TNF-α, PCT, CRP, etc.—to aid sepsis detection and improve recognition rates [24,25,26,27]. While valuable as references, these biomarkers lack pathogen specificity. Current mainstream POCT systems for bloodstream infections, such as FilmArray BCID and BioFire, are primarily designed for use with positive blood culture samples rather than direct detection of the whole blood. Therefore, while these systems provide rapid identification after culture positivity, they do not eliminate the time required for the initial blood culture step, which remains a key limitation in early diagnosis [28].

The proposed OM-NPCR-POCT assay in this study comprises three pairs of outer primers, inner primers, and three TaqMan probes. After sample pretreatment, specimens are loaded into cartridges for instrument-based detection. The process occurs in two stages: during the first stage, outer primers amplify and enrich DNA templates; subsequently, by altering annealing temperatures, inner primers further amplify the enriched DNA templates. Probes release detectable fluorescent signals during this amplification. The simultaneous presence of inner and outer primers within the OM-NPCR system ensures high specificity. To reduce the risk of contamination, the PCR system employs a premixed reagent containing dUTP/UDG to prevent cross-contamination. In a series of sensitivity comparison experiments, OM-NPCR consistently demonstrated higher detection sensitivity than conventional PCR, regardless of whether plasmid templates, nucleic acids, or clinical samples processed by different methods (including routine manual extraction and POCT integrated extraction) were used. This fully demonstrates that the OM-NPCR method possesses superior detection sensitivity. The ENucFlow-PS2 system employed in this study integrates ultrasonic lysis, magnetic bead-based nucleic acid extraction, qPCR amplification, and 7-color fluorescence detection. It effectively degrades the cell walls of Gram-positive bacteria (such as *Streptococcus pneumoniae*) [29], enhancing nucleic acid extraction efficiency. Rapid temperature cycling shortens fluorescence detection time, with the overall testing duration ≤ 2 h. In terms of cost, the compact device design (448 mm × 178 mm × 331 mm) and room-temperature-stable reagents reduce transportation expenses. Technical integration and fully enclosed testing chamber design eliminate aerosol leakage risks, eliminating the need for expensive PCR lab construction (no strict zoning or purification systems required) or large automated instruments, resulting in low infrastructure demands. The platform delivers test results within approximately two hours, enabling physicians to initiate precise treatment earlier, reduce hospital stays and related complications, and lower overall healthcare expenditures. This achieves cost reductions across multiple dimensions, including equipment, space, and time. Rapid deployment is suitable for primary-care hospitals and resource-constrained areas.

In blood PCR testing, the most critical issue is inhibitor suppression. Hemoglobin compounds in blood are considered major PCR inhibitors, interfering with DNA polymerase activity [30]. Direct addition of large blood volumes to POCT reduces detection rates. To address this, we employed a simple pretreatment method to remove blood inhibitors, followed by a combined approach of heating with nucleic acid release agents and centrifugation for crude cell lysis as shown in Section 2.7. This facilitated red blood cell lysis, hemoglobin precipitation, and protein digestion. While using patient plasma may mitigate inhibitor interference, the absence of components like white blood cells may lead to bacterial loss, resulting in lower PCR positivity rates compared to whole blood testing [31]. Our preliminary testing confirmed this. Numerous studies have also proposed detection methods, such as using an ultra-high-speed, highly sensitive nanomechanical sensor array to detect EDTA whole blood samples from blood culture-confirmed bacteremia patients, identifying *Escherichia coli* and *Staphylococcus aureus* with a detection limit of 20 CFU/mL [32]. Meanwhile, the detection limit for *Clostridium difficile* using qPCR was 88.56 copies/reaction [33]. This study systematically evaluated and proposed a method with higher sensitivity: OM-NPCR achieved a limit of detection (LOD) as low as 1–4 copies/μL for pure plasmids of ECO, KPN, and SPN. In simulated clinical blood samples, OM-NPCR-POCT achieved detection limits of 10–20 CFU/mL for these three pathogens, demonstrating higher sensitivity than qPCR.

This study has several limitations. First, the method is primarily qualitative and can only reflect changes in bacterial load based on the relative differences in amplification signals, achieving relative quantification. We hope that, with the advancement of nested PCR, standardized frameworks for quantitative interpretation will be developed in the future, which would improve the ability to distinguish between contamination and infection. Second, like all PCR technologies, detection of bacterial DNA does not necessarily indicate the presence of viable bacteria. Despite implementing multiple contamination control measures, residual DNA from non-viable bacteria may still affect the results, and positive findings should be interpreted in conjunction with clinical context. Third, larger cohort studies are needed in the future to further validate the performance of this assay for SPN. Finally, as this assay currently targets only three common bloodstream pathogens, its diagnostic scope is limited. Therefore, this assay must be used in parallel with conventional blood culture techniques to ensure that other pathogens are not overlooked. In addition, due to instrument limitations, the present study employed a standard thermal cycling protocol rather than a rapid cycling program, which limited further reduction in the overall turnaround time. Future optimization under rapid cycling conditions may further shorten the assay duration while maintaining performance. The modular design of this detection system allows expansion of the range of detectable pathogens. We plan to incorporate a broader panel of pathogens in future studies to better support clinical diagnosis.

## 5. Conclusions

In summary, we have developed a rapid, sensitive, and accurate OM-NPCR-POCT detection method, providing a novel approach for the direct detection of *Escherichia coli*, *Klebsiella pneumoniae*, and *Streptococcus pneumoniae* in whole blood.

## Figures and Tables

**Table 1:** Primer and probe sequences used in OM-NPCR.

Strain (Gene)	Primer/Probe	Sequence (5′-3′)	Source
ECO (ybbw)	ECO-OF	CGGGGTCACTGGCCTGCTTGATTCTGATTGG	This study
	ECO-OR	CCGACCAGTGAAATCGCCCAAATCGCCATACC	This study
	ECO-IF	GCAAAATCTGGCCGGGAT	[15]
	ECO-IR	AATCGCCCAAATCGCCA	[15]
	ECO-P ^a^	CY5-CACTGCCATTCTTAACCCGTGCATC-BHQ2	[15]
KPN (khe)	KPN-OF	CGCACCTACGTCTCAACCGGCTGGGGAT	This study
	KPN-OR	CGCCCACCACCAGCAGACGAACTTCCTG	This study
	KPN-IF	CTGGGGATCCACCACGAG	[16]
	KPN-IR	AGCTTCCAGAGATAGCCGTTTAT	[16]
	KPN-P ^a^	FAM-AGGAAGAGTTCATCTACGTGCTGGAGG-BHQ1	[16]
SPN (lytA)	SPN-OF	TGAGACCTATGCAGCGGTTGAACTGATTGAAAGCCATT	This study
	SPN-OR	TTAAACTGCTCACGGCTAATGCCCCATTTTGCCAAGTA	This study
	SPN-IF	ACGCAATCTAGCAGATGAAGCA	[17]
	SPN-IR	TCGTGCGTTTTAATTCCAGCT	[17]
	SPN-P ^a^	VIC-GCCGAAAACGCTTGATACAGGGAG-BHQ1	[17]

Note. ^a^ Probe modifications: FAM, 6-carboxyfuorescein; VIC, 6-phosphoramidite; CY5, Cyanine 5; BHQ, black hole quencher; ECO, *Escherichia coli*; KPN, *Klebsiella pneumoniae*; SPN, *Streptococcus pneumoniae*.

**Table 2:** Detection of ECO, KPN, and SPN in Clinical Samples.

Species	OM-NPCR-POCT			qPCR			Kappa Value
	Positive	Negative	Positive Accuracy Rate(%)	Positive	Negative	Positive Accuracy Rate(%)	
ECO	14	76	70	12	78	60	0.910
KPN	16	74	80	13	77	65	0.877
SPN	4	96	80	3	97	60	0.852

Note. The number of positive and negative samples represents the total number of blood samples tested using OM-NPCR and qPCR. ECO, *Escherichia coli*; KPN, *Klebsiella pneumoniae*; SPN, *Streptococcus pneumoniae*.

## Data Availability

The original contributions presented in the study are included in the article/Supplementary Materials, further inquiries can be directed to the corresponding authors.

## Supplementary Materials

The following supporting information can be downloaded at: https://www.mdpi.com/article/10.3390/pathogens15020211/s1, Table S1: The reproducibility of OM-NPCR; Table S2: Specificity Testing.
