# Supplementary material for: A Nested PCR-Based Point of Care Testing of Multiplex Pathogens Associated with Bloodstream Infection
Source: Pathogens. 2026 Feb 13;15(2):211. doi: 10.3390/pathogens15020211 (PMC12943058; doi:10.3390/pathogens15020211)
Supplement: Supplementary file 1 [file pathogens-15-00211-s001.zip › pathogens-4117248-supplementary.pdf]

Table S1. The reproducibility of OM-NPCR

Standard DNA(copies/μL)	ECO	KPN	SPN
10^5	8/8	8/8	8/8
10^4	8/8	8/8	8/8
10^3	8/8	8/8	8/8
10^2	8/8	8/8	8/8
10^1	8/8	8/8	8/8
5	8/8	8/8	8/8
10^0	2/8	6/8	7/8

ECO, *Escherichia coli*; KPN, *Klebsiella pneumoniae*; SPN, *Streptococcus pneumoniae*.

Table S2: Specificity Testing

Strains	Origin	ECO	KPN	SPN
Escherichia coli	ATCC 25922	Positive	Negative	Negative
Klebsiella pneumoniae	ATCC 11296	Negative	Positive	Negative
Streptococcus pneumoniae	ATCC 49619	Negative	Negative	Positive
Streptococcus agalactiae	ATCC 13813	Negative	Negative	Negative
Streptococcus pyogenes	ATCC 19615	Negative	Negative	Negative
Listeria monocytogenes	ATCC 19111	Negative	Negative	Negative
Staphylococcus aureus	ATCC 29213	Negative	Negative	Negative
Pseudomonas aeruginosa	ATCC 27853	Negative	Negative	Negative
Neisseria meningitidis	Isolated	Negative	Negative	Negative
Enterococcus faecium	Isolated	Negative	Negative	Negative
Enterococcus faecalis	Isolated	Negative	Negative	Negative
Enterobacter cloacae	Isolated	Negative	Negative	Negative
Proteus mirabilis	Isolated	Negative	Negative	Negative
Pseudomonas maltophilia	Isolated	Negative	Negative	Negative
Candida tropicalis	ATCC 750	Negative	Negative	Negative
Candida krusei	ATCC 6258	Negative	Negative	Negative
Candida glabrata	ATCC 2001	Negative	Negative	Negative
Candida albicans	ATCC 753	Negative	Negative	Negative

ECO, *Escherichia coli*; KPN, *Klebsiella pneumoniae*; SPN, *Streptococcus pneumoniae*.
